# Multi-domain-fusion deep learning for automatic modulation recognition in spatial cognitive radio

**DOI:** 10.1038/s41598-023-37165-2

**Published:** 2023-07-03

**Authors:** Shunhu Hou, Yaoyao Dong, Yuhai Li, Qingqing Yan, Mengtao Wang, Shengliang Fang

**Affiliations:** 1 Graduate School, Space Engineering University, Beijing, 101416 China; 2 School of Space Information, Space Engineering University, Beijing, 101416 China; 3 Xichang Satellite Launch Center, Xichang, 615000 China

**Keywords:** Aerospace engineering, Electrical and electronic engineering, Computational science, Information technology

## Abstract

Automatic modulation recognition (AMR) is a critical technology in spatial cognitive radio (SCR), and building high-performance AMR model can achieve high classification accuracy of signals. AMR is a classification problem essentially, and deep learning has achieved excellent performance in various classification tasks. In recent years, joint recognition of multiple networks has become increasingly popular. In complex wireless environments, there are multiple signal types and diversity of characteristics between different signals. Also, the existence of multiple interference in wireless environment makes the signal characteristics more complex. It is difficult for a single network to accurately extract the unique features of all signals and achieve accurate classification. So, this article proposes a time–frequency domain joint recognition model that combines two deep learning networks (DLNs), to achieve higher accuracy AMR. A DLN named MCLDNN (multi-channel convolutional long short-term deep neural network) is trained on samples composed of in-phase and quadrature component (IQ) signals, to distinguish modulation modes that are relatively easy to identify. This paper proposes a BiGRU3 (three-layer bidirectional gated recurrent unit) network based on FFT as the second DLN. For signals with significant similarity in the time domain and significant differences in the frequency domain that are difficult to distinguish by the former DLN, such as AM-DSB and WBFM, FFT (Fast Fourier Transform) is used to obtain frequency domain amplitude and phase (FDAP) information. Experiments have shown that the BiGUR3 network has superior extraction performance for amplitude spectrum and phase spectrum features. Experiments are conducted on two publicly available datasets, the RML2016.10a and RML2016.10b, and the results show that the overall recognition accuracy of the proposed joint model reaches 94.94% and 96.69%, respectively. Compared to a single network, the recognition accuracy is significantly improved. At the same time, the recognition accuracy of AM-DSB and WBFM signals has been improved by 17% and 18.2%, respectively.

## Introduction

In recent years, the role of communication technology in the 5G era has become increasingly apparent. Spatial cognitive radio technology is particularly essential^1,2^. This requires that information processing technologies based on artificial intelligence also evolve and progress^3^. Among them, automatic modulation recognition(AMR) has an actual application in wireless communication information processing^4^. However, many signal identification research studies are based on a single network, resulting in many signals that cannot be effectively distinguished. References^5–13^ propose single network identification algorithms, which are advanced, but all suffer from signal confusion problems. The literatures^14–17^ propose improved single networks, which still have signal confusion problems, although the overall recognition accuracy has been improved. It is difficult for these single networks to accurately extract the unique features of all signals and achieve accurate classification. Multi-domain fusion processing and joint modulation recognition methods are used relatively infrequently.

The traditional AMR methods are mainly divided into likelihood based (LB) AMR^18,19^ and feature based (FB) AMR^20–22^. LB AMR obtains the optimal modulation estimation by minimizing the probability of misclassification. This method has the drawbacks of high computational complexity and narrow applicability. FB AMR distinguishes different types of modulation signals based on their characteristics. Such as wavelet spectral features, cyclic spectral features, and high-order spectral features.

Deep learning has been very hot in recent years^23^ and is increasingly used in the field of AMR^24^. Neural networks have become a hot spot for research. Traditional pattern recognition methods^25^ require a lot of labor cost in extracting signal features, and at the same time the classification accuracy of signals is not satisfactory^26^. However, neural networks can automatically extract features from a large number of signal samples^27^, solving the drawbacks of pattern recognition^28^.

O'Shea created the RML2016.10a dataset in 2016, introducing convolutional neural network(CNN) into the field of AMR^29^, and achieved a signal classification accuracy of 79.3% on this dataset. The following year, Nathan E. West applied the CLDNN network model to the field of AMR^30^, and the recognition accuracy reached 83.3% on the RML2016.10a dataset. Fuxin Zhang proposed the PET-CGDNN network using CNN and gated recurrent units (GRUs) as feature extraction layers in reference^5^. The network maintains a recognition accuracy of over 90%. Although these networks have achieved good recognition levels, they still cannot distinguish between QAM16 and QAM64 signals, as well as AM-DSB and WBFM.

Yu Wang et al.^31^ proposed a data-driven fusion model which combines two CNN networks, one trained on the IQ signal dataset, and the other trained on the constellation map dataset. This provides a joint scheme to distinguish between QAM16 and QAM64. Mengtao Wang^32^ proposed a joint automatic modulation recognition method combining deep learning and expert features to distinguish QAM16 from QAM64 with high accuracy, and improve the overall network recognition performance in spatial cognitive communication. However, WBFM and AM-DSB signals are still confused.

Jialang Xu proposed a spatiotemporal multi-channel learning framework (MCLDNN) for automatic modulation recognition^6^. This network could extract features more effectively from the perspective of time and space. The network has distinguished well between QAM16 and QAM64 at high SNRs, but more than half of the WBFM signals are still misidentified as AM-DSB. This limits the overall recognition accuracy of the network. In our previous study, there was also the problem of difficult distinction between WBFM and AM-DSB signals^14^.

Therefore, in this paper, we propose a time–frequency domain joint recognition model that combines two deep learning networks, the MCLDNN and BiGRU3, to achieve higher accuracy AMR. The joint model is practical and can be used in contemporary wireless communication systems. The main innovation points are as follows.

1. Propose a multi-domain fusion-based deep learning framework combining two neural networks. One DLN is used to classify IQ signals, and the other DLN is used to classify FDAP signals. The FDAP signals are obtained by FFT of IQ signals that are difficult to distinguish in the former DLN. In this way, form a joint AMR model.
2. Built a novel deep learning network, BiGRU3, that can accurately extract amplitude spectrum and phase spectrum features in the frequency domain.
3. Introduce FFT to obtain amplitude and phase feature information of AM-DSB and WBFM in the frequency domain. And form a new two-class dataset called DW based on the amplitude and phase characteristics. At the same time, BiGRU3 is used to classify DW, and the accuracy is improved by 17% and 18.2% on the two publicly available datasets, respectively, compared to the IQ sequence classification of the two types of signals using MCLDNN.
4. Verify the rationality and practicability of the multi-domain-fusion deep learning framework on two published datasets. Using the RML2016.10a and RML2016.10b datasets, we establish joint recognition model for the similarity of AM-DSB and WBFM signals in the time domain, respectively. Experiments show that the joint model has good recognition performance on both datasets. The overall recognition accuracy can reach 94.94% and 96.69%, respectively. It is progressiveness in the current AMR field.

## System model

### Intelligent receiver system based on zero-IF architecture in spatial cognitive radio

**Figure 1:**
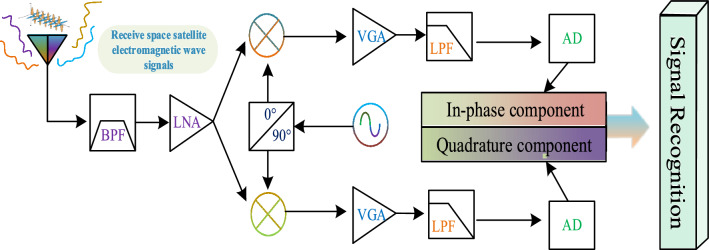
Quadrature sampling zero-IF intelligent receiver.

Quadrature sampling is the most common receiver architecture^33^, where the IQ signal is sampled to obtain a data stream. In this paper, this architecture will be used for network design. Figure 1 lists the quadrature sampling form of the zero-IF receiver. Each sample obtains both I and Q signals with dimension N × 2, where N corresponds to the time length of the signal and 2 corresponds to the in-phase component I and the quadrature component Q. The workflow of this intelligent satellite communication receiver is as follows: the RF signal first passes through the mid-pass filter BPF and the low-noise amplifier LNA for frequency selection and amplification. Then the signal is fed into the mixer and the local oscillator frequency for mixing to produce the in-phase component I and quadrature component Q. Then the I and Q signals are amplified, filtered, sampled and extracted to form a digital IQ baseband signal. Finally, the acquired IQ baseband signal is fed into the AMC model to complete the identification of the signal modulation type.

### The joint AMC model

**Figure 2:**
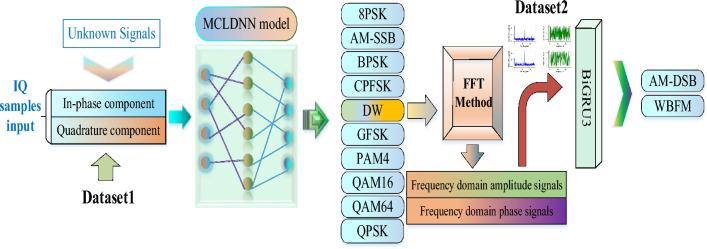
The joint AMC model.

This paper proposes a multi-domain fusion-based deep learning framework combining two neural networks. As shown in Fig. 2, using the joint model to identify multiple modulated signals widely used in modern wireless communication systems. When the receiver acquires the unknown signals, the first stage will be made by MCLDNN to identify them. In addition, AM-DSB and WBFM are considered the same class and named DW in this stage. In the second stage, using FFT to construct frequency domain sampling features, establishing a new dataset, and then feeding it into the BiGRU3 network for binary classification recognition of DW.

## Method of proposing models

### MCLDNN network

**Figure 3:**
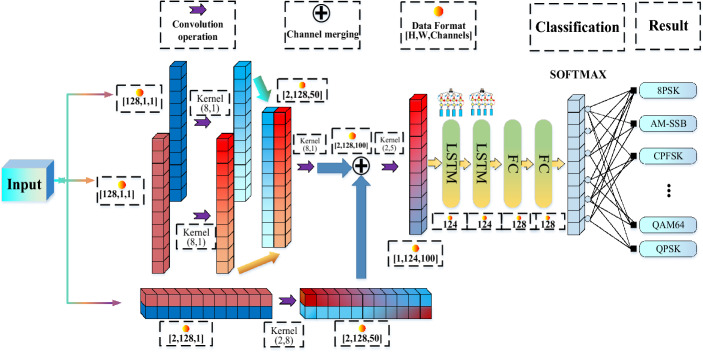
The MCLDNN model structure.

MCLDNN is a novel three-stream deep learning framework to extract the features from individual and combined in-phase/quadrature (I/Q) symbols of the modulated data^6^. The proposed framework integrates one-dimensional (1D) convolutional, two-dimensional (2D) convolutional and long short-term memory (LSTM) layers to extract features more effectively from a time and space perspective. The network structure diagram is shown in Fig. 3.

Figure 3 shows that the network divides the input IQ signals into three channels and extracts the time sequence features of the route I, route Q, and route IQ, respectively. The IQ data input form during model training is [Batch_size, 2, 128, channels], which belongs to four-dimensional data. MCLDNN networks are spliced in the channels dimension when merging channels. Then, using the convolution kernel of (2,5) for further feature extraction. The extracted features are input into the two-layer LSTM to extract the time sequence feature further, and connecting the two-layer DNN for classification.

### BiGRU3 network

**Figure 4:**
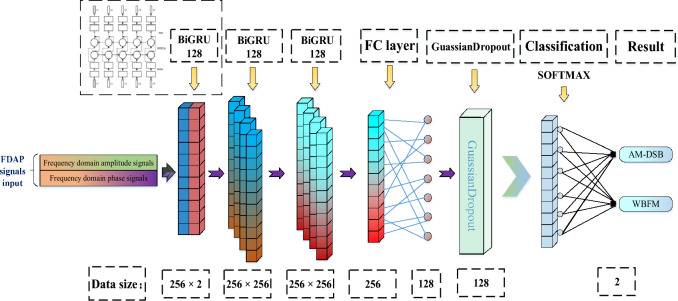
The BiGRU3 model structure.

GRU is a highly effective variant of LSTM networks, with a simpler structure and better performance compared to LSTM networks. Therefore, it is currently a very popular neural network. GRU has excellent extraction ability for sequence data features and can solve long-term dependency problem. Dehua Hong et al. proposed the GRU2 network in reference^34^, demonstrating that GRU has good feature extraction performance for time-domain amplitude spectrum and phase spectrum features, with a classification accuracy of 91.3% for multiple signals. Therefore, we propose a BiGRU3 network that extracts frequency domain amplitude and phase features. This network is composed of three layers of BiGRU, which more fully extracts data features. We add a layer of fully connected network (FC) and a layer of GuassianDropout to combine the features and implement the classification. The network structure of BiGRU3 is shown in Fig. 4.

The specific parameters of the BiGRU3 network are shown in the following Table 1:

**Table 1:** The structure of the BiGRU3 network.

Layer type	Input size	Output size	Details
Input	256 × 2	256 × 2	–
BiGRU_1	256 × 2	256 × 256	return_sequence = True
BiGRU_2	256 × 256	256 × 256	return_sequence = True
BiGRU_3	256 × 256	256	return_sequence = False
FC	256	128	Units:128
GuassianDropout	128	128	Random Gaussian noise
Classify + Softmax	128	2	One-Hot Output

### Discrete Fourier transform and fast Fourier transform

Discrete Fourier transform (DFT) is a form of Fourier transform that takes a discrete form in both the time and frequency domains. It is capable of transforming a sample of time domain signals into a sample in the discrete time Fourier transform (DTFT) frequency domain^35,36^.

The process of transforming a discrete signal $x(n)$ of length $L$ into a spectrum $\{ X(k)\}_{k = 0}^{N - 1}$ of length $N(N \ge L)$ is called the discrete Fourier transform (DFT):

1 $$ X(k) = \sum\limits_{n = 0}^{N - 1} {x(n)e^{ - j2\pi kn/N} } , \, k = 0,1,...,N - 1 $$

$X(k)$ must be a complex sequence and $X(k)$ satisfies conjugate symmetry with respect to $X(N/2)$, as shown in Eq. (2).

2 $$ X(k) = X^{*} (N - k)\quad (k = 1,2, \ldots ,N/2 - 1) $$

Set the modulus and phase of $X(k)$ to $\left| {X(k)} \right|$ and $\theta (k)$, respectively. The Inverse Discrete Fourier Transform (IDFT) of $X(k)$ is shown in Eq. (3).

3 $$ \begin{aligned} IDFT[X(k)] & = x(n) = \frac{1}{N}\sum\limits_{k = 0}^{N - 1} X (k){\text{e}}^{{j\frac{2\pi }{N}kn}} \\ & = \frac{1}{N}\sum\limits_{k = 0}^{N - 1} | X(k)|{\text{e}}^{j\theta (k)} {\text{e}}^{{j\frac{2\pi }{N}kn}} \\ & = \frac{1}{N}\sum\limits_{k = 0}^{N - 1} | X(k)|\cos \left[ {\frac{2\pi }{N}kn + \theta (k)} \right] \\ \end{aligned} $$

Combining Eqs. (1) and (3), the amplitude of $X(k)$ in the frequency domain is shown in Eq. (4).

4 $$ \left| {X\left( k \right)} \right| = \left\{ {\begin{array}{*{20}l} \begin{gathered} \left| {\frac{1}{N}\sum\limits_{n = 0}^{N - 1} x (n)e^{{ - j\frac{2\pi }{N}kn}} } \right|(k = 0 \, or \, N/2) \hfill \\ \hfill \\ \end{gathered} \hfill \\ {\left| {\frac{2}{N}\sum\limits_{n = 0}^{N - 1} x (n)e^{{ - j\frac{2\pi }{N}kn}} } \right|(k = 1,2, \cdots ,N/2 - 1)} \hfill \\ \end{array} } \right. $$

Set $$A(k)$$:

5 $$ A(k) = \left\{ {\begin{array}{*{20}l} \begin{gathered} \frac{1}{N}\sum\limits_{n = 0}^{N - 1} x (n)e^{{ - j\frac{2\pi }{N}kn}} (k = 0 \, or \, N/2) \hfill \\ \hfill \\ \end{gathered} \hfill \\ {\frac{2}{N}\sum\limits_{n = 0}^{N - 1} x (n)e^{{ - j\frac{2\pi }{N}kn}} (k = 1,2, \cdots ,N/2 - 1)} \hfill \\ \end{array} } \right. $$

The phase of $X(k)$ in the frequency domain is shown in Eq. (6).

6 $$ \theta (k) = \arctan \left(\frac{{{\text{Im}} [A(k)]}}{{{\text{Re}} [A(k)]}}\right) $$

According to the DFT principle above, the output $X(k)$ is the value of the sample point in the frequency domain, and the value is in the form of a complex number. The format is as follows:

7 $$ X(k) = [a_{1} + jb_{1} ,a_{2} + jb_{2} ,...,a_{k} + jb_{k} ], \, k = 1,...,N $$

Therefore, further calculate the amplitude $S_{k}$ and phase $\Phi_{k}$ of the signal in the frequency domain. As shown in Eqs. (8, 9).

8 $$ S_{k} = \left| {X(k)} \right| = \sqrt {a_{k}^{2} + b_{k}^{2} } , \, k = 1,2,...,N $$

9 $$ \Phi_{k} = \theta (k) = \arctan (\frac{{b_{k} }}{{a_{k} }}), \, k = 1,2,...,N $$

The Fast Fourier Transform (FFT) is a fast algorithm for the discrete Fourier transform^37,38^. The basic idea is to take the original N-point sequence and decompose it into a series of short sequences in turn. By making full use of the symmetric and periodic properties of the exponential factors in the DFT computational equation, the corresponding DFTs of these short sequences are then derived and appropriately combined to remove repeated calculations, reduce multiplication operations and simplify the structure. The results of both transforms are consistent, but the FFT can significantly reduce the running time.

The discrete values in the time domain can be converted into discrete values of magnitude and phase in the frequency domain by the FFT transform. For AM-DSB and WBFM signals, the time domain expression of the amplitude modulation signal (AM) is shown in Eq. (10).

10 $$ {\text{S}}_{AM} (t) = [A_{0} + m(t)]\cos \omega_{c} t $$

where $A_{0}$ is the applied DC(Direct Current) component, m(t) is the deterministic signal, which can also be a random signal, and $\omega_{c}$ is the carrier frequency.

All the information in the AM signal is transmitted through the sideband, and the DC component does not carry information. To improve the modulation efficiency and power utilization, the DC component is removed from the AM modulation model. That is, a signal with a high efficiency modulation model, called AM-DSB, can be obtained. The expression in the time domain is shown in Eq. (11).

11 $$ {\text{S}}_{DSB} (t) = m(t)\cos \omega_{c} t $$

And the WBFM signal time domain expression is shown below.

12 $$ {\text{S}}_{WBFM} (t) = A\cos (\omega_{c} t + K_{FM} \int {f(t)dt} ) $$

where $K_{FM} \int {f(t)dt}$ is the FM coefficient and $f(t)$ is the modulated signal. When $|K_{FM} \int {f(t)dt} |_{MAX} > > \frac{\pi }{6}$ is a wideband FM signal (WBFM).

From the time domain expressions of the two signals, it can be seen that the two have high similarity in the time domain waveforms. So it is not easy to distinguish them by the time domain sampling points. The WBFM signal is a wideband FM signal with a different frequency component from the AM-DSB signal in the frequency domain. This way, we can obtain the two signals' sampling points in the frequency domain through FFT transform. Theoretically, the two signals can be distinguished with higher accuracy by this.

Assume that the input IQ two-way time domain sample point data is $in\_samples$.

13 $$ in\_samples = \left[ {\begin{array}{*{20}c} {\begin{array}{*{20}c} {x_{I1} } & {x_{I2} } & {...} & {x_{Ii} } \\ \end{array} } \\ {\begin{array}{*{20}c} {x_{Q1} } & {x_{Q2} } & {...} & {x_{Qi} } \\ \end{array} } \\ \end{array} } \right] \, i = 1,2,...,N $$

After FFT, the frequency domain amplitude and phase information of the signal are obtained. As shown in formulas (14) and (15).

14 $$ S_{FDA} = \left[ {\begin{array}{*{20}c} {\begin{array}{*{20}c} {s_{I1} } & {s_{I2} } & {...} & {s_{Ii} } \\ \end{array} } \\ {\begin{array}{*{20}c} {s_{Q1} } & {s_{Q2} } & {...} & {s_{Qi} } \\ \end{array} } \\ \end{array} } \right] \, i = 1,2,...,N $$

15 $$ \Phi_{FDP} = \left[ {\begin{array}{*{20}c} {\begin{array}{*{20}c} {\varphi_{I1} } & {\varphi_{I2} } & {...} & {\varphi_{Ii} } \\ \end{array} } \\ {\begin{array}{*{20}c} {\varphi_{Q1} } & {\varphi_{Q2} } & {...} & {\varphi_{Qi} } \\ \end{array} } \\ \end{array} } \right] \, i = 1,2,...,N $$

The binary classification experiment in this paper wants to combine the frequency domain amplitude information with the phase information into the BiGRU3 network, so the new input data format is obtained as follows.

16 $$ i\tilde{n} = vstack[hstack(S^{(1)}_{FDA} ,S^{(2)}_{FDA} ), \, hstack(\Phi^{(1)}_{FDP} ,\Phi^{(2)}_{FDP} )] $$

In the formula (16), $hstack$ represents horizontal consolidation, $vstack$ represents vertical consolidation, and $S^{(i)}_{FDA}$ represents the i-th row of data in $S_{FDA}$.

## Experimental results

### MCLDNN decile experiments

#### The RML2016.10a dataset

This paper uses a popular open-source dataset RML2016.10a^39^. This dataset has 11 classes of modulated signals with SNR ranging from − 20 to 18 dB, and the length of a single sample is 128. Each signal is IQ two-way, and the data format is [128,2]. The details are shown in Table 2.

**Table 2:** The RML2016.10a dataset.

Dataset	RML2016.10a
Modulation types	8 Digital Modulations: BPSK, QPSK, 8PSK, 16QAM,64QAM, BFSK, CPFSK, and PAM4 3 Analog Modulations: WBFM, AM-SSB, and AM-DSB
Signal format	In-phase and quadrature (IQ) [128,2]
SNR range	[− 20 dB, − 18 dB, . . . , 18 dB]
Number of single class	20,000
Total number of samples	220,000

**Figure 5:**
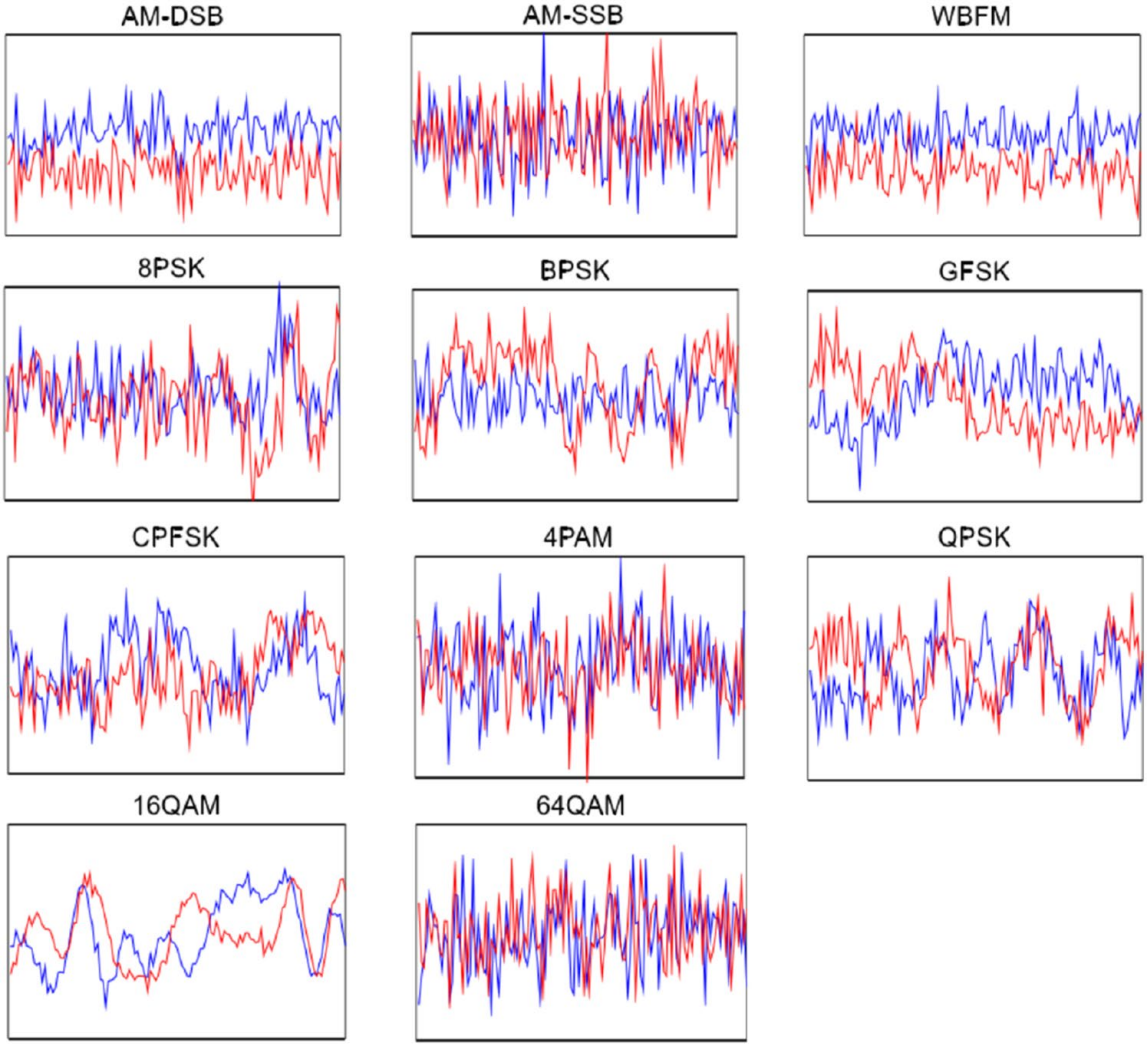
Time-domain waveforms of 11 modulated signals.

Figure 5 shows the time domain waveforms of each modulation mode in the dataset, where each image is a randomly selected sample of the corresponding modulation mode.

In Fig. 5, each type of signal is divided into IQ channels. The red line is the I signal, and the blue line is the Q signal. The horizontal axis of each type of signal graph is the time axis, and the vertical axis is the amplitude of the signal. We can visually observe from Fig. 5 that the time domain waveform plots of AM-DSB and WBFM signals have a large similarity. Therefore, inputting the time-domain sampling points into the neural network tends to cause the feature blurring problem and makes it difficult to distinguish between these two types of signals.

#### Experimental parameters setting

All experimental parameters are set the same. 60% of the total data set is randomly selected as the training set, 20% as the validation set, and 20% as the test set. The Epoch size is 50, and Batch_size is set to 128. Using the Adam optimizer based on stochastic gradient descent, and choosing the cross-entropy loss function. The initial learning rate is 0.001, and the learning rate is halved for every 10 Epochs trained. The detailed training parameters are shown in Table 3.

**Table 3:** Training parameters.

Training parameters	MCLDNN
Dataset partitioning ratio	6:2:2
Batch_size	128
Epoch	50
Optimizer	Adam
Loss function	Cross entropy
Initial learning rate	0.001
Classification function	Softmax

#### The effectiveness of MCLDNN network

**Figure 6:**
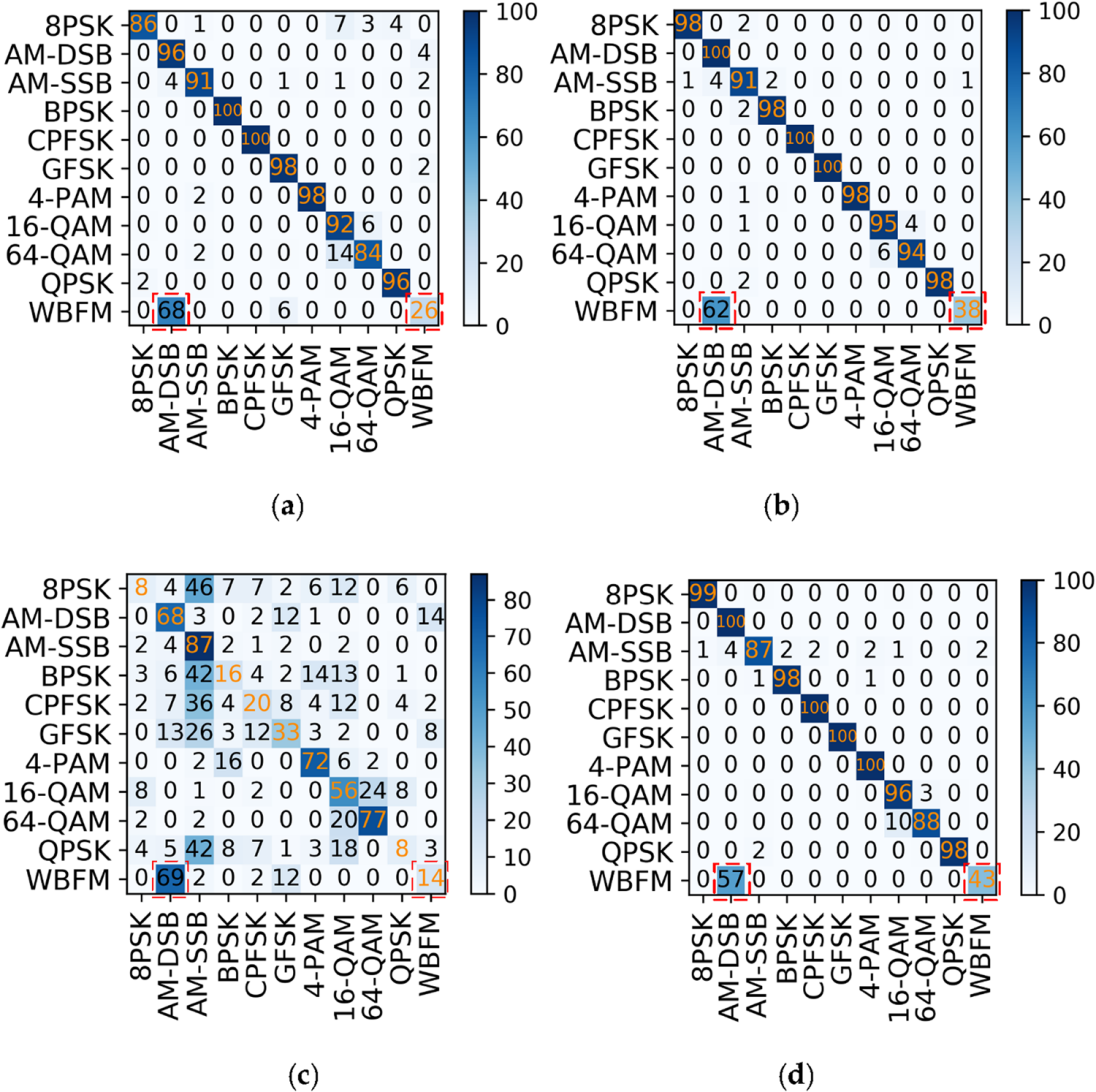
Confusion matrices of MCLDNN at different SNRs on the RML2016.10a dataset. (**a**) SNR = 0 dB; (**b**) SNR = 8 dB; (**c**) SNR = -8 dB; (**d**) SNR = 18 dB.

The 11 classes of modulated signals on the RML2016.10a dataset are identified using the MCLDNN network, and a partial confusion matrices of the identification results are shown in Fig. 6.

In a confusion matrix, the accuracy of the recognition result is expressed by the color depth of each square. The abscissa of the confusion matrix represents the results of classifying various modulated signals with networks. The ordinate represents the true modulation methods. If the diagonal of the matrix is darker, then the model’s prediction accuracy is better. If the color blocks are scattered throughout the matrix and are not concentrated at the diagonal, then the recognition effect is not ideal. Figure 6 shows that using the MCLDNN network to identify the 11 types of signals with good recognition accuracy at high SNRs. However, AM-DSB and WBFM signals are seriously confused. As shown in the red box in the figure, more than half of the WBFM signals are still mistaken for AM-DSB, even under the high SNRs condition.

**Figure 7:**
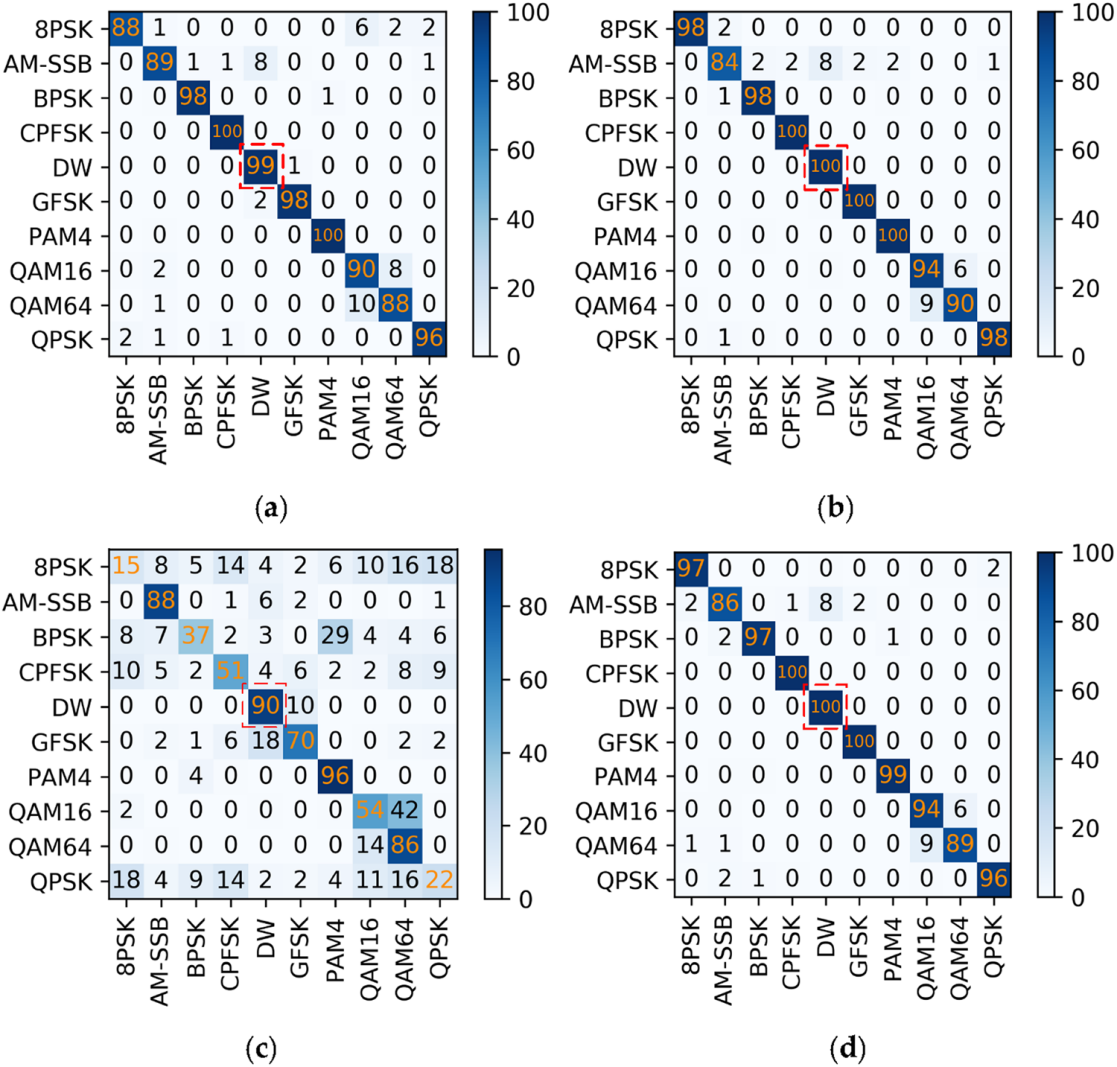
Confusion matrices of MCLDNN at different SNRs in decile experiment. (**a**) SNR = 0 dB; (**b**) SNR = 8 dB; (**c**) SNR = -8 dB; (**d**) SNR = 18 dB.

For this problem, a joint modulation identification scheme is used. The AM-DSB and WBFM signals are combined and named DW signals. The original 11 class signals are integrated into 10 class signals and sent to the MCLDNN network for identification. The identification results are shown in Fig. 7.

**Figure 8:**
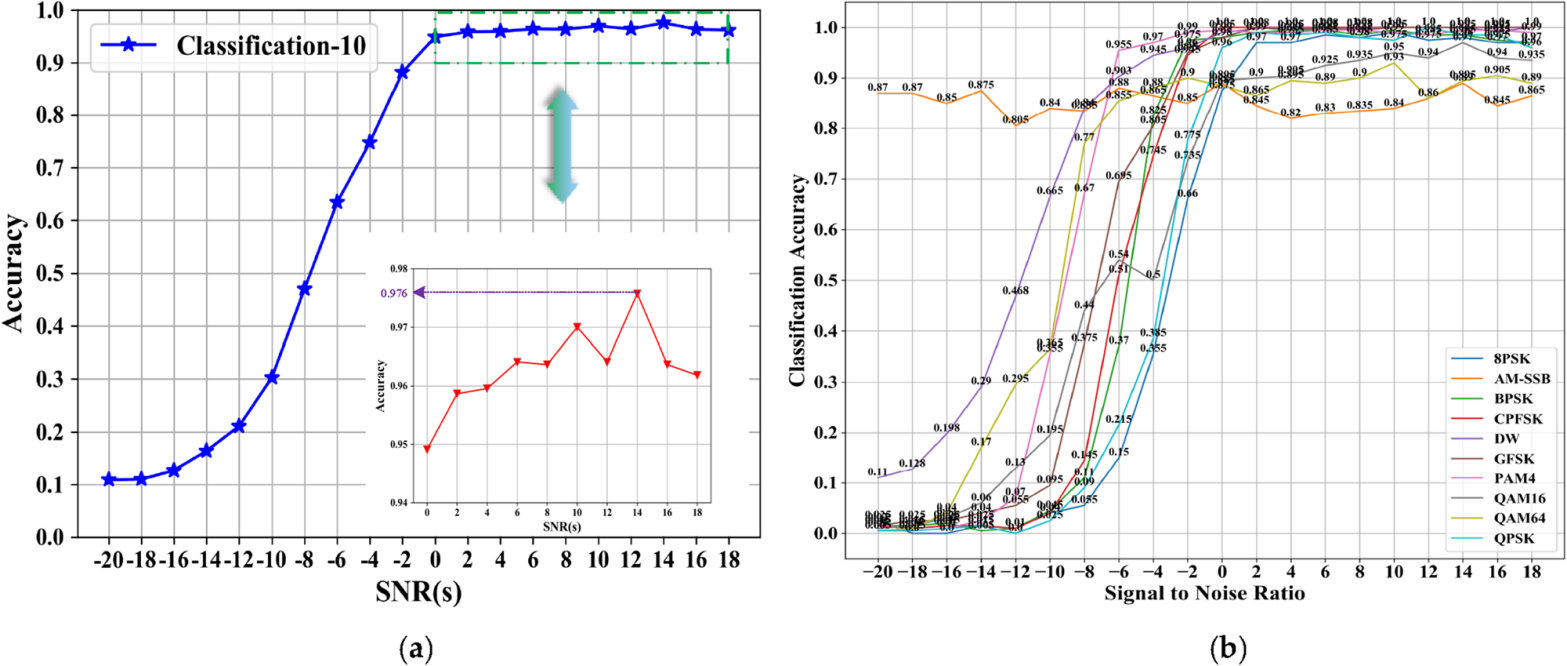
Decile accuracy chart. (**a**) Overall recognition rates; (**b**) accuracy for each mod.

The 10-classification experiment distinguishes the DW signals from the other 9 categories well. Even if the SNR is − 8 dB, there is still a high degree of differentiation. Meanwhile, the recognition accuracy of the MCLDNN network for 10-class signals also reaches an outstanding level. It reaches 97.6% at a SNR of 14 dB, and the average recognition accuracy reaches 96.3% at a SNR greater than 0 dB. The recognition accuracy graph is shown in Fig. 8. Figure 8b represents the accuracy of each type of signal at different SNR conditions. From the figure, the DW signal can reach 100% recognition accuracy under high SNR condition. It indicates that the influence of other signals on the DW signal is very small. In this way, the DW signal can be subsequently separated on the basis of ten-class experiments for binary classification experiments on the frequency domain.

### Second classification experiment

Based on the results of the decile experiments in "MCLDNN Decile Experiments" section, using the FFT to sample the AM-DSB and WBFM signals in the frequency domain for DW signals. The amplitude and phase information of the frequency domain sampling are input into the BiGRU3 network for the bifurcation task. The detailed binary classification dataset is shown in Table 4.

**Table 4:** Second classification dataset.

Second classification dataset	DW
Modulation types	2 Analog modulations: WBFM and AM-DSB
Signal format	Frequency domain amplitude and phase signals(FDAP)
Signal size	[256,2]
SNR range	[− 20 dB, − 18 dB, . . . , 18 dB]
Number of single class	20,000
Total number of samples	40,000

When generating DW dataset, comparing the FFT transformation and DFT transformation, it is found that the FFT is much lower than the DFT in terms of time cost. This proves the superiority and rationality of using FFT transformation in this paper. See Table 5 for detailed information.

**Table 5:** Comparison table of FFT and DFT on the RML2016.10a dataset.

DW (RML2016.10a)	DFT	FFT
Modulation types	WBFM, AM-DSB	WBFM, AM-DSB
Signal format	IQ [128,2]	IQ [128,2]
The converted data format	FDAP [256,2]	FDAP [256,2]
Number of signals	40,000	40,000
Transform time	9080 s	2.3 s

**Figure 9:**
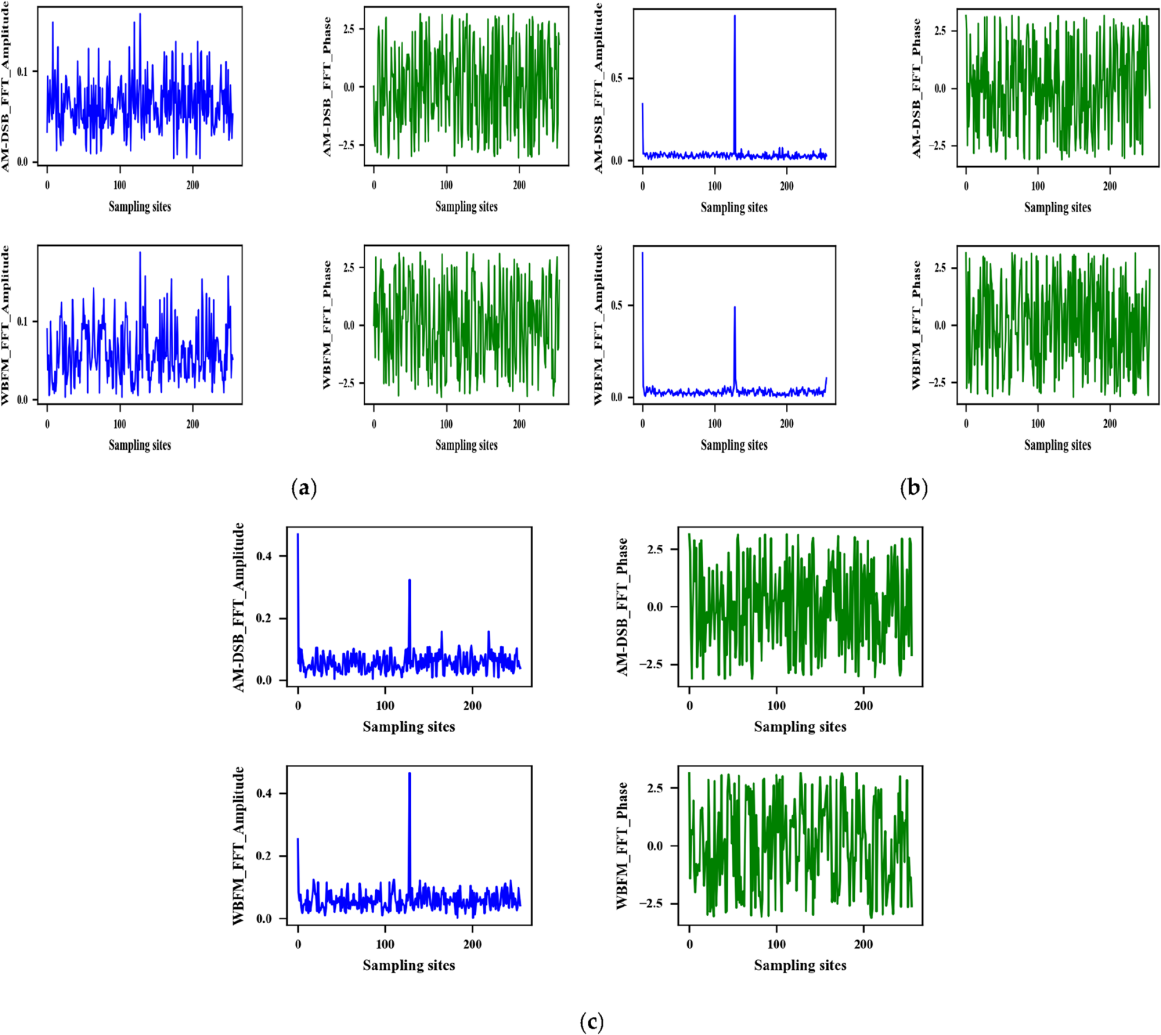
Frequency domain sampling comparison chart. (**a**) SNR = − 10 dB; (**b**) SNR = 8 dB; (**c**) SNR = 0 dB.

To facilitate the comparison of the difference between the two types of signals in the frequency domain, the frequency domain amplitude and phase sampling plots of the two types of signals are taken here when the SNR is − 10 dB, 0 dB, and 8 dB, respectively. This is shown in Fig. 9.

**Figure 10:**
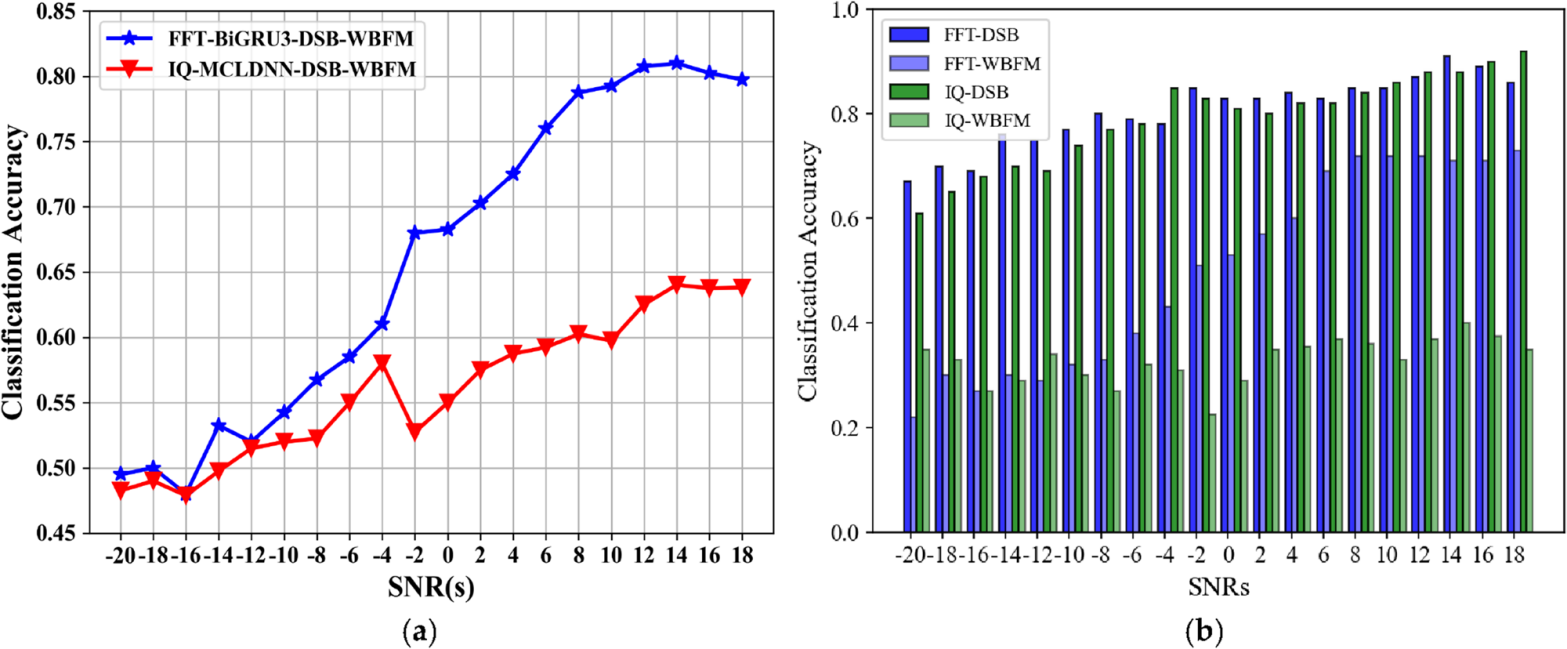
Second classification experiment result. (**a**) Recognition accuracy of IQ signal and FFT signal, respectively; (**b**) time–frequency binary classification accuracy comparison chart.

As shown in Fig. 9, the amplitude and phase characteristics of AM-DSB and WBFM signals in the frequency domain have pronounced differences. The DW dataset is input into the BiGRU3 network for classification, while the time-domain IQ signal binary classification dataset is input into the MCLDNN network for classification. Compare the classification accuracy of the two methods. Figure 10a plots the overall recognition accuracy of the two datasets with different SNRs. Figure 10b plots the respective accuracy of AM-DSB and WBFM for the two datasets at different SNRs.

**Figure 11:**
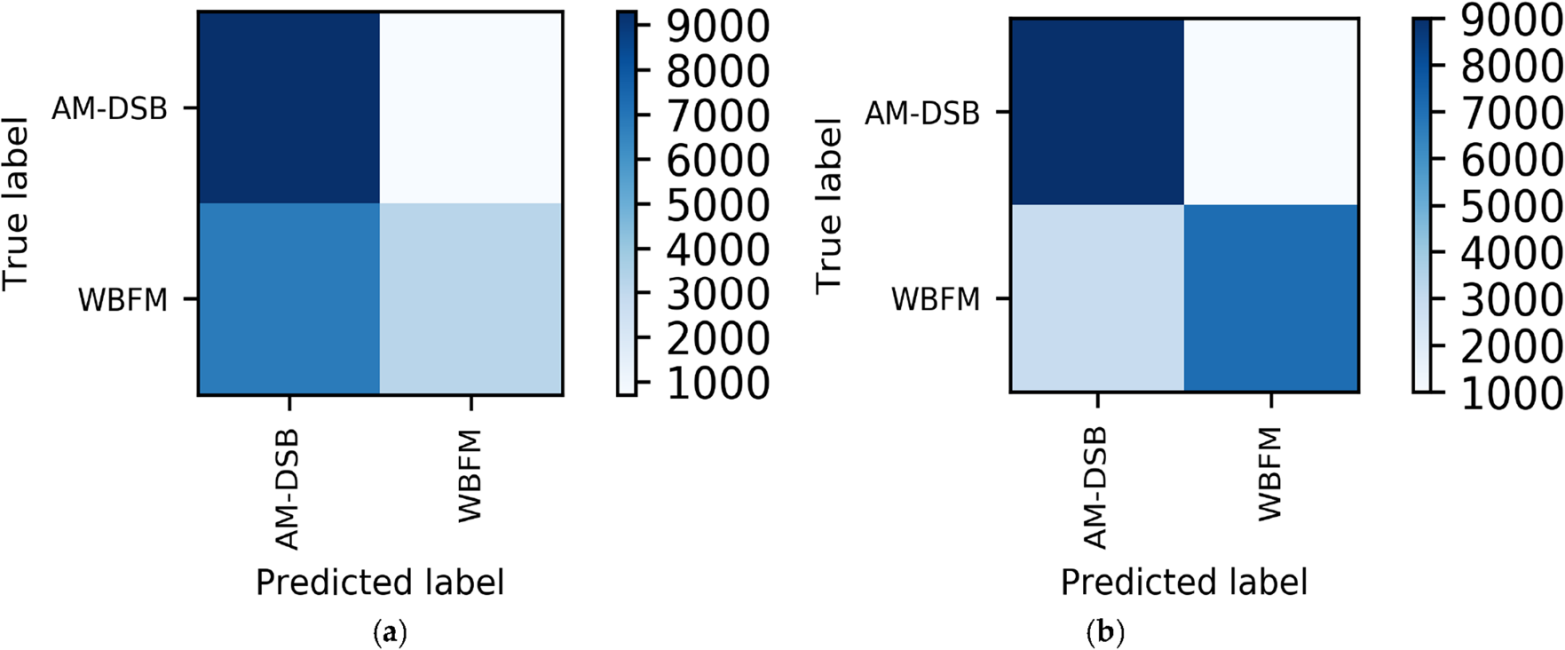
Time–frequency binary classification confusion matrix accuracy comparison charts (SNR = 8 dB). (**a**) IQ signals binary classification result by MCLDNN; (**b**) FFT signals binary classification result by BiGRU3.

From the comparison graph of the binary classification results of the time domain and frequency domain, we can obtain that the frequency domain recognition results can reach 81% accuracy when the SNR is 14 dB, and the average recognition accuracy can reach 76.7% when the SNR is greater than 0 dB. Compared with the IQ signal dataset input into the MCLDNN model, the binary classification results are improved by 17% and 16.3% by the BiGRU3 model, respectively. The confusion matrix when taking the SNR of 8 dB is shown in Fig. 11. Figure 11 shows that more than half of the WBFM signals are misidentified as AM-DSB in the bifurcation results of the time-domain IQ signals. However, the bifurcation results of the frequency-domain sampled signals show that the frequency-domain features can better distinguish the two types of signals.

### The joint model results

In "Second classification experiment" section, the AM-DSB and WBFM signals are sampled in the frequency domain by performing the Fast Fourier Transform to extract the frequency domain amplitude and phase feature information. By combining the decile model in "MCLDNN decile experiments" section, a joint model can be established to identify and classify 11 types of signals automatically. At the same time, the recognition accuracy of each class of signals in the joint model at each SNR can be easily deduced. The three networks in Table 6 are selected as the comparison models. CNN^40^ is the first classical structure that uses a convolutional neural network to recognize modulation. CLDNN^30^ is a classical structure in speech recognition tasks that has been successfully transplanted into the field of electromagnetic signal information processing. CLDNN2 is a convolutional long short-term deep neural network proposed by Xiaoyu Liu^13^, which deepens the depth of convolutional layers and increases the number of convolutional kernels based on CLDNN.

**Table 6:** Structural parameters of comparison networks.

Models	CNN	CLDNN	CLDNN2
Convolution layers	2	3	4
Kernel size	(1,2) (2,3)	(1,8)	(1,3) (2,3) (1,3) (1,3)
Convolution channels	256, 80	50,50,50	256, 256, 80, 80
LSTM layers	0	1	1
LSTM units	0	50	50

In addition to the CNN networks mentioned above, Ade Pitra Hermawan proposed the IC-AMCNet network^8^. Compared with the existing CNN architecture, it has adjusted the number of layers and added new layer types to meet the estimated delay standards beyond the fifth generation (B5G) communication. Njoku proposed the CGDNet network^9^ and introduced the GuassianDropout, which enhanced the feature extraction process and prevented the problem of gradient vanishing. FuxinZhang proposed a PET-CGDNN network based on phase parameter estimation and transformation^5^. Compared with CLDNN network and CLDNN2 network, this network uses CNN and GRU as feature extraction layers, greatly reducing training parameters. Rajendran proposed the LSTM2 network^7^, which converts IQ data into (amplitude-phase)AP data, achieving high recognition accuracy with a simple network structure. The structures of all comparison networks are shown in Table 7 below.

**Table 7:** Network structures of the comparison models.

Model	Reference	Input	Main structure
CNN	Reference^40^	I/Q	CNN
CLDNN	Reference^30^	I/Q	CNN + LSTM + DNN
CLDNN2	Reference^13^	I/Q	CNN + LSTM + DNN
LSTM2	Reference^7^	Amplitude/Phase	2 LSTM layers
MCLDNN	Reference^6^	I/Q, I and Q	Multi-channel CNN + LSTM + DNN
IC-AMCNet	Reference^8^	I/Q	CNN + Gaussian noise
CGDNet	Reference^9^	I/Q	CNN + GRU + DNN
PET-CGDNN	Reference^5^	I/Q	CNN + GRU + DNN

**Figure 12:**
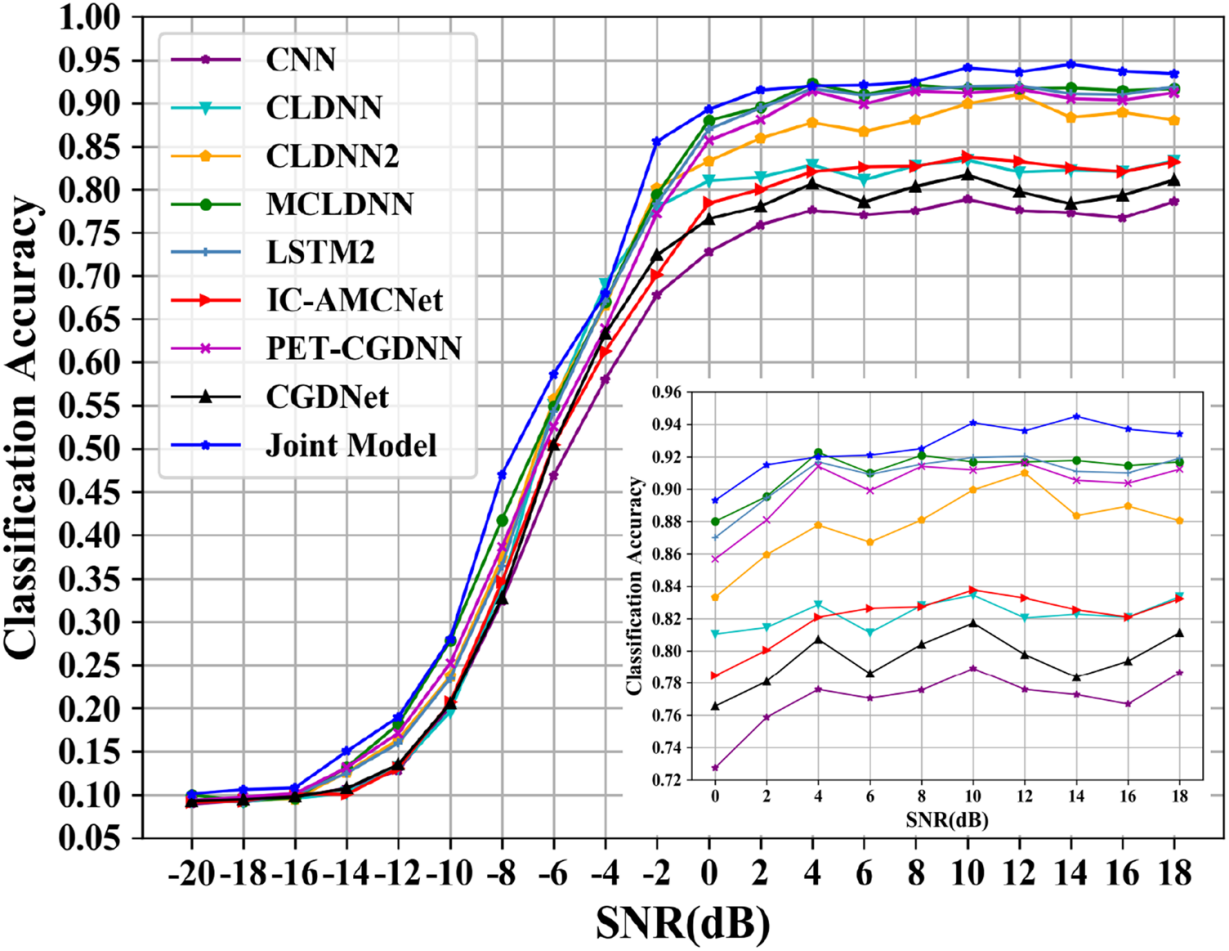
Classification accuracy of different networks on the RML2016.10a dataset.

The classification accuracies of all models are shown in Fig. 12. The joint recognition model has better recognition results under low and high SNR conditions. In the SNR range, the highest recognition accuracy of the CNN network can reach 79.1%; the highest recognition accuracy of the CLDNN network can reach 83.7%; the CLDNN2 network can achieve 90.8% recognition rate; the LSTM2 network can achieve 92.05% recognition rate; the CGDNet network can achieve 81.72% recognition rate; the IC-AMCNet network can achieve 83.77% recognition rate; the PET-CGDNN network can achieve 91.6% recognition rate. The MCLDNN network can achieve 92.27% recognition accuracy, and the average recognition accuracy can reach 91.12% when the SNR is greater than 0 dB. The joint model can achieve 94.94% recognition accuracy, and the average recognition accuracy can reach 93.07% when the SNR is greater than 0 dB. Compared with the MCLDNN network, the overall recognition accuracy has been significantly improved.

**Figure 13:**
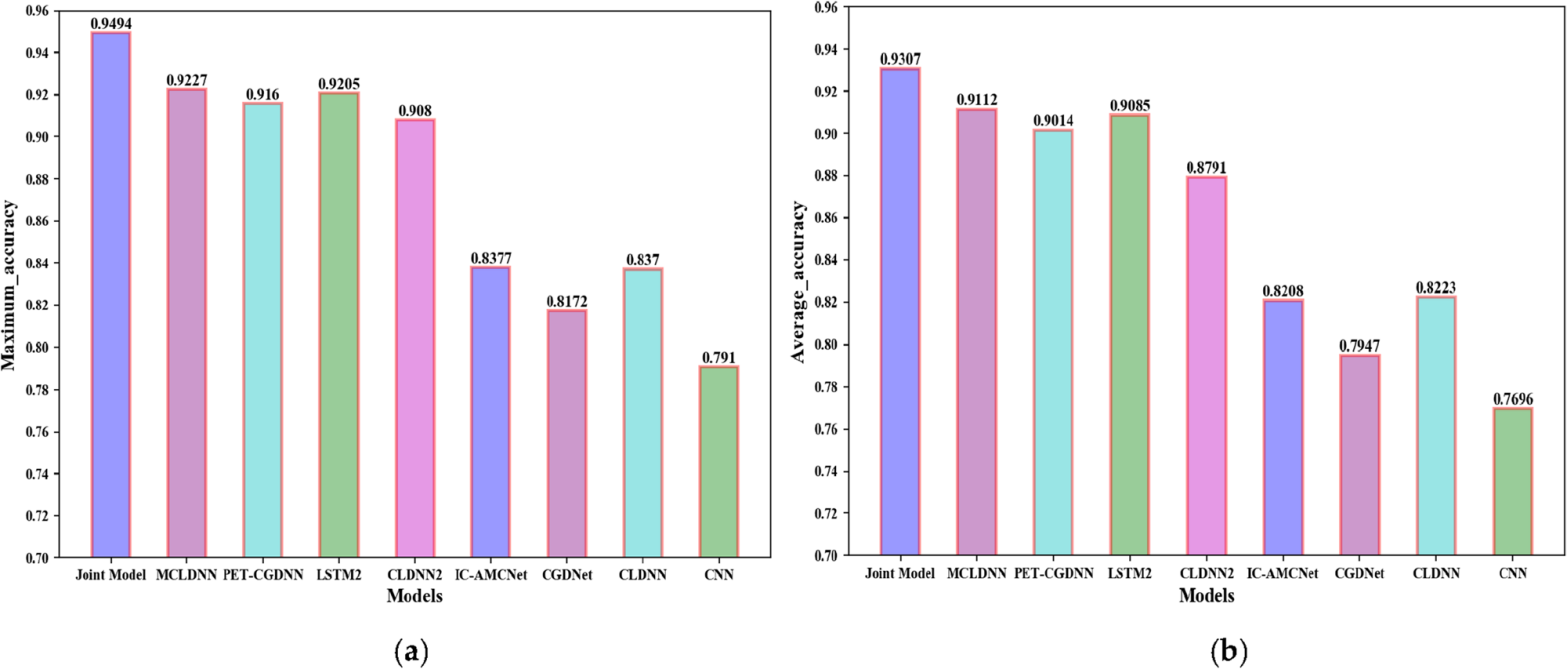
Accuracy comparison chart of the nine models on the RML2016.10a dataset. (**a**) The maximum accuracy; (**b**) The average accuracy.

The recognition accuracies obtained by the nine networks are visually represented by a histogram, as shown in Fig. 13. The maximum accuracy represents the maximum recognition accuracy in the SNR range. The average accuracy represents the average recognition accuracy when the SNR is greater than 0 dB.

**Figure 14:**
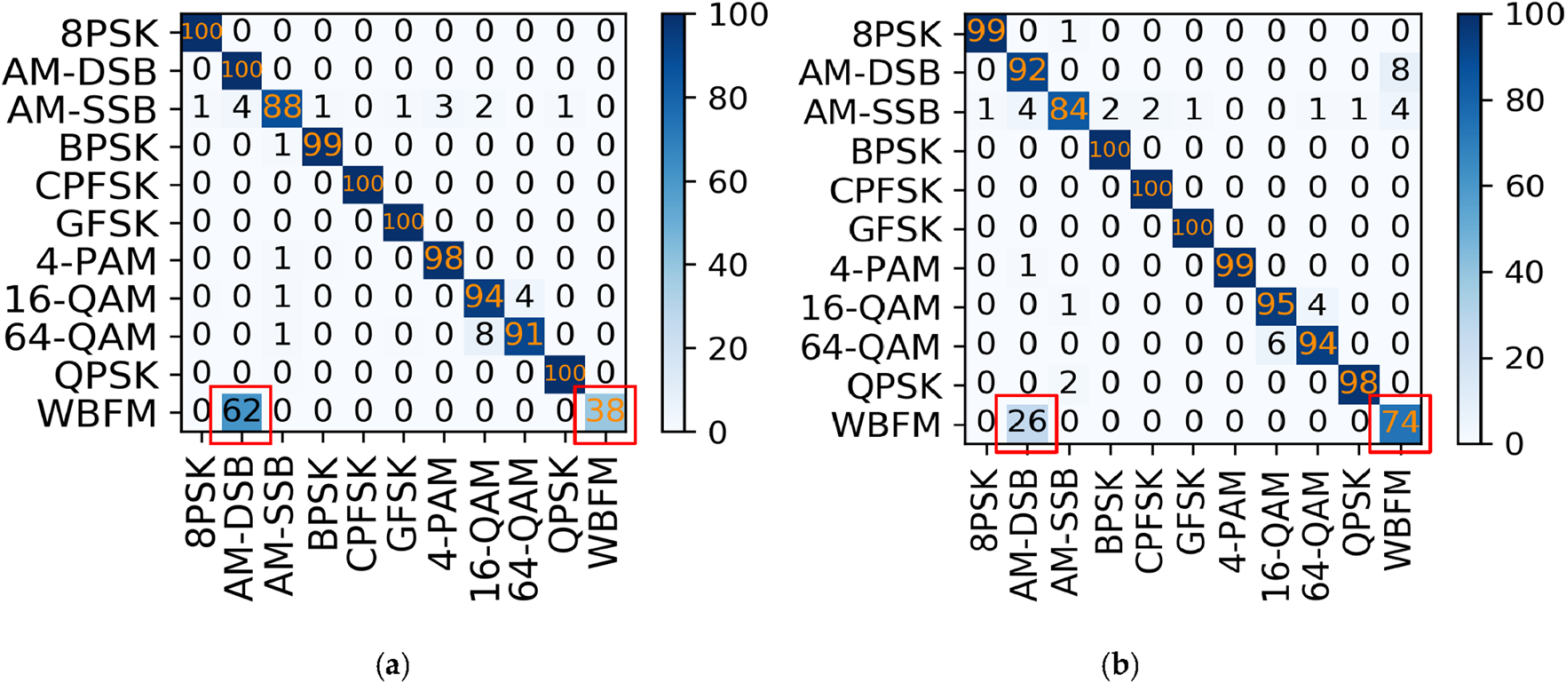
Confusion matrices of the two methods when SNR is 10 dB. (**a**) MCLDNN network; (**b**) The joint model.

From Fig. 13, we can find that the recognition accuracy of the joint model is significantly better than the existing benchmark networks. In addition, we provide the confusion matrices of MCLDNN and the Joint model at 10 dB in Fig. 14a,b. It can be seen that the Joint model significantly improves the recognition ability of AM-DSB and WBFM.

### The RML2016.10b dataset experiment

#### The RML2016.10b dataset

The above experiment solves the problem that AM-DSB and WBFM signals are difficult to distinguish in the time domain for RML2016.10a dataset, and improves the recognition performance of the overall model. In this section, use another dataset to verify the performance of the joint signal recognition model. The RML2016.10b dataset has 10 types of modulated signals^41^, which is one class less than the RML2016.10a dataset, but the RML2016.10b signal count is larger. Details are shown in Table 8.

**Table 8:** The RML2016.10b dataset.

Dataset	RML2016.10b
Modulation types	8 digital modulations: BPSK, QPSK, 8PSK, 16QAM,64QAM, BFSK, CPFSK, and PAM4 2 analog modulations: WBFM and AM-DSB
Signal format	In-phase and quadrature (IQ) [128,2]
SNR range	[− 20 dB, − 18 dB, . . . , 18 dB]
Number of single class	120,000
Total number of samples	1,200,000

#### Benchmark network experiment

**Figure 15:**
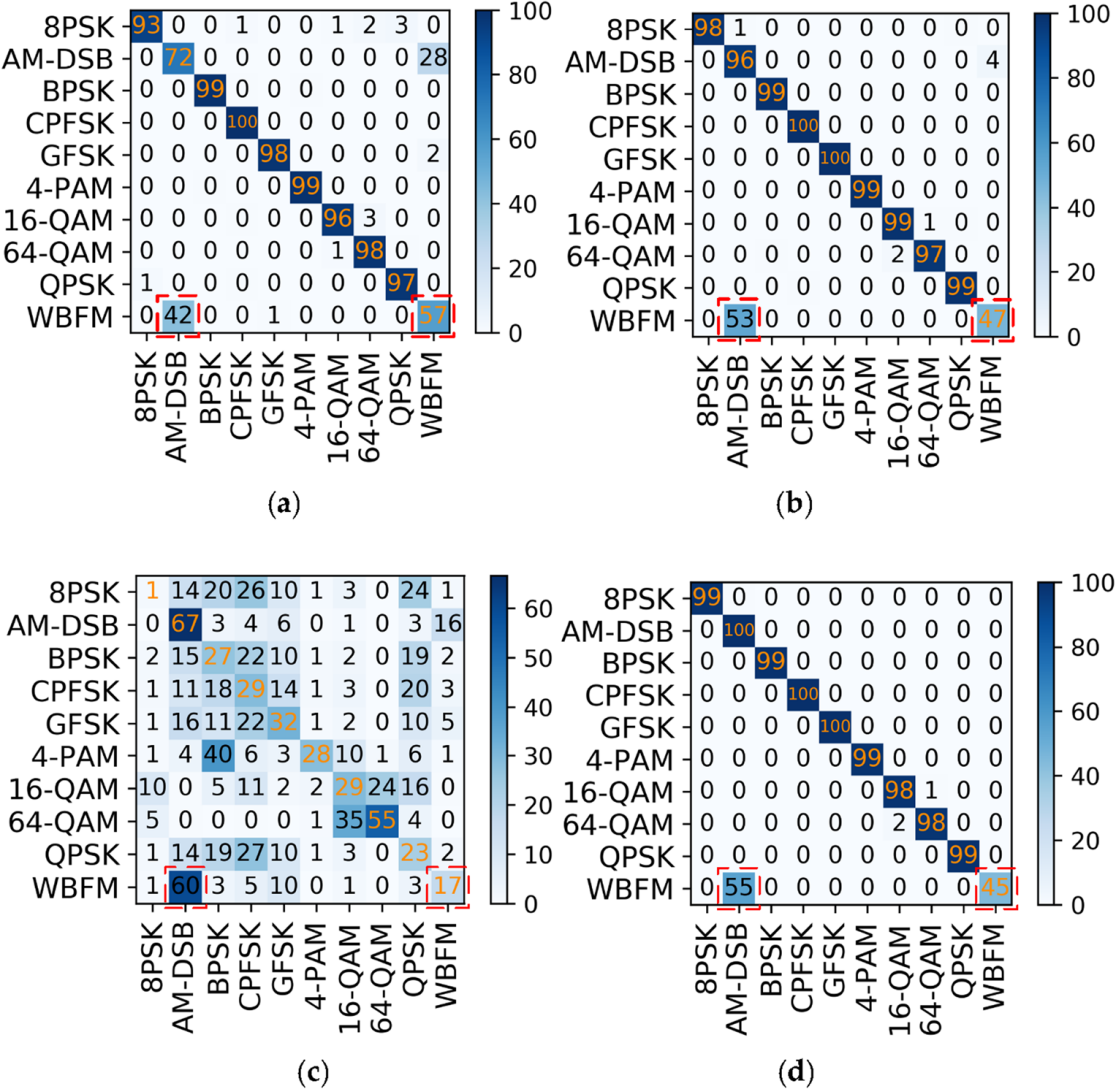
Confusion matrices of MCLDNN at different SNRs on the RML2016.10b dataset. (**a**) SNR = 0 dB; (**b**) SNR = 8 dB; (**c**) SNR = -8 dB; (**d**) SNR = 18 dB.

Use the MCLDNN network as the benchmark network to identify the ten types of signals in RML2016.10b. The experimental results are shown in Fig. 15.

From Fig. 15, the AM-DSB and WBFM signals in the RML2016.10b dataset are seriously confused, which affects the overall recognition rate.

#### Joint model experiment

Establish a joint signal recognition model for the RML2016.10b dataset. We combine AM-DSB and WBFM signals into a class of signals DW. Continue to identify and classify with other 8 types of signals to obtain high classification accuracy of various types of signals. Then perform FFT transformation on the DW dataset, extract amplitude and phase features in the frequency domain, and perform a binary classification experiment. The details of the DW dataset are shown in Table 9 below.

**Table 9:** Comparison table of FFT and DFT on the RML2016.10b dataset.

DW (RML2016.10b)	DFT	FFT
Modulation types	WBFM, AM-DSB	WBFM, AM-DSB
Signal format	IQ [128,2]	IQ [128,2]
The converted data format	FDAP [256,2]	FDAP [256,2]
Number of signals	240,000	240,000
Transform time	15.13 h	13.9 s

**Figure 16:**
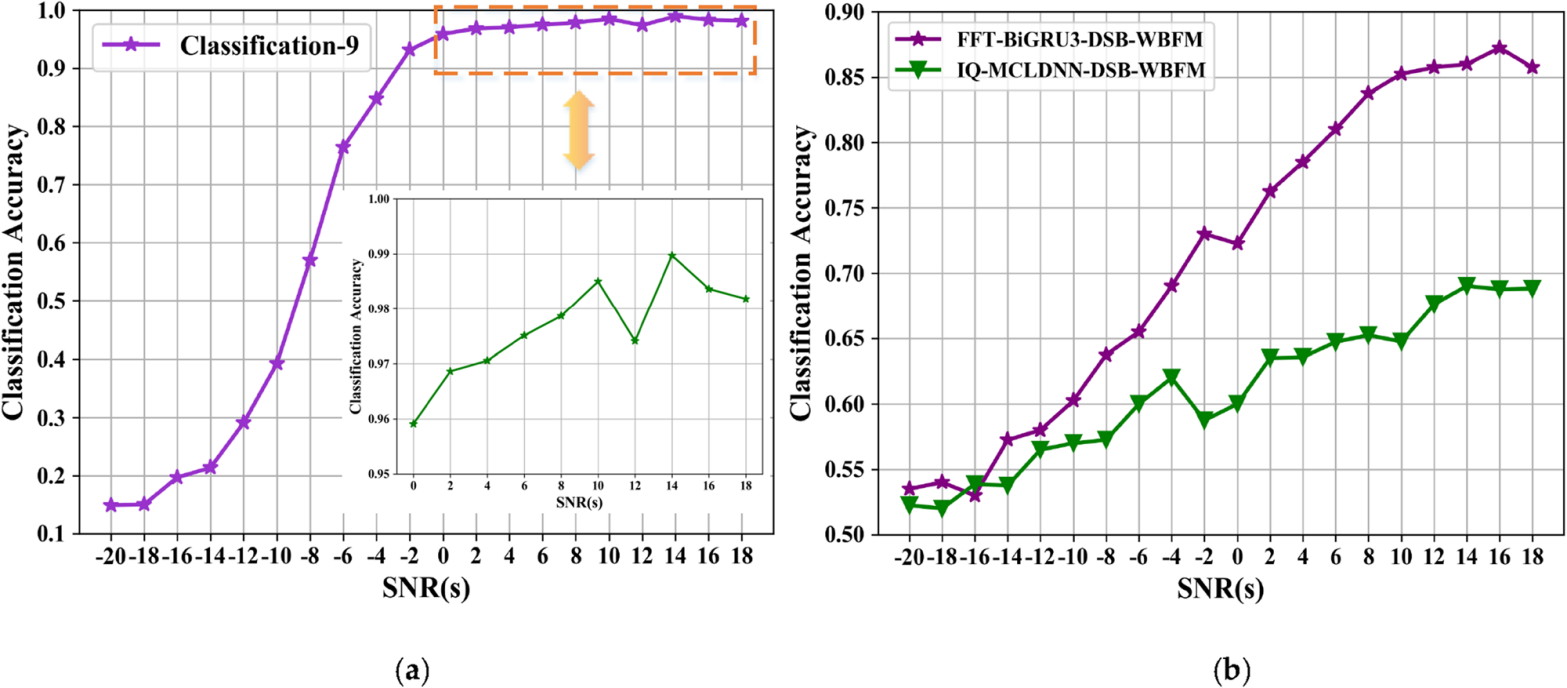
Step by step results of the joint model on the RML2016.10b dataset. (**a**) Nine classification experiment result; (**b**) Second classification experiment result.

Input DW and other 8 types of signals into the MCLDNN network for nine classification experiment, and perform two classification experiment on the DW dataset by the BiGRU3 network. The results are shown in Fig. 16. We find that after FFT transformation, the binary classification accuracy of AM-DSB and WBFM is improved by 18.2%.

**Figure 17:**
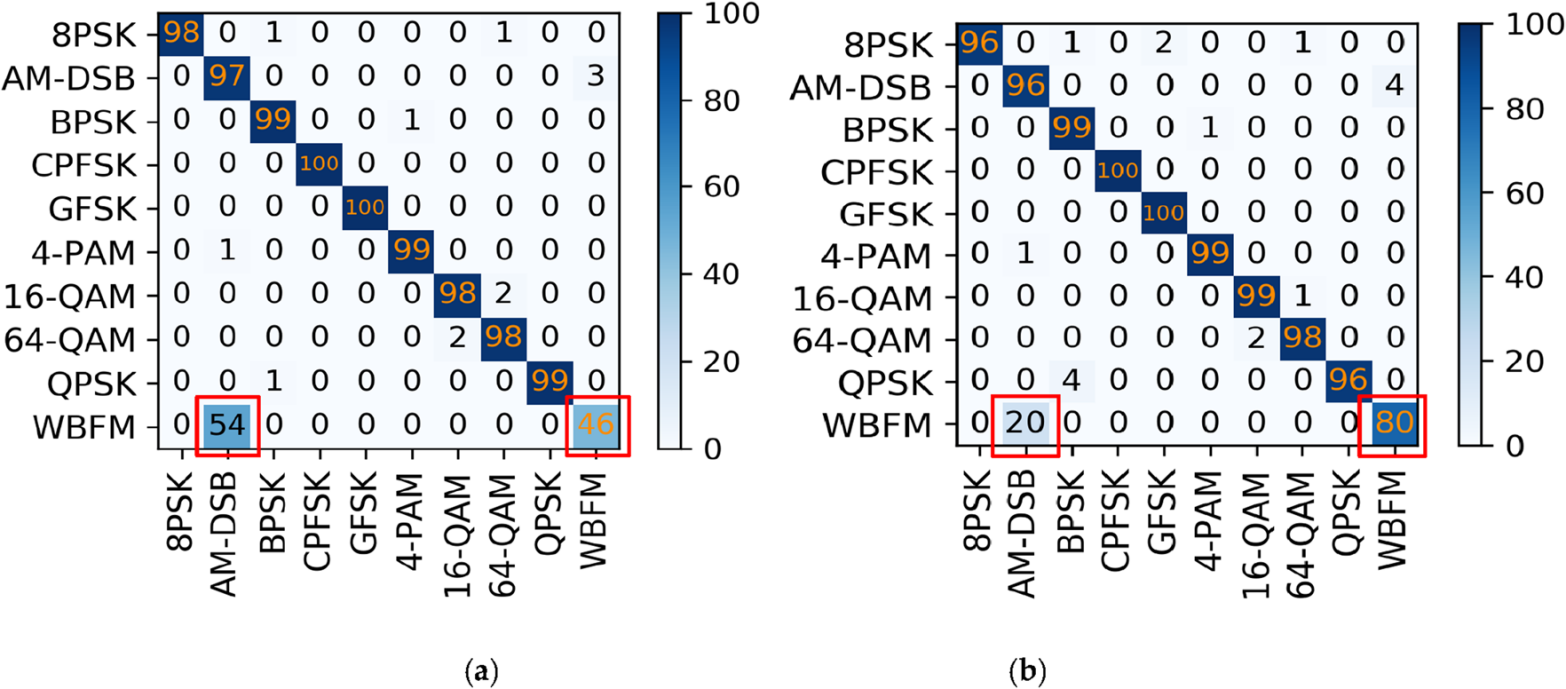
Confusion matrices of the two methods when SNR is 10 dB. (**a**) MCLDNN network; (**b**) The joint model.

In this way, we can easily obtain the overall recognition accuracy of the model. Select the confusion matrices when the SNR is 10 dB. As shown in Fig. 17. For the RML2016.10b dataset, the joint recognition model also effectively improves the confusion between AM-DSB and WBFM signals, and improves their recognition accuracy.

**Figure 18:**
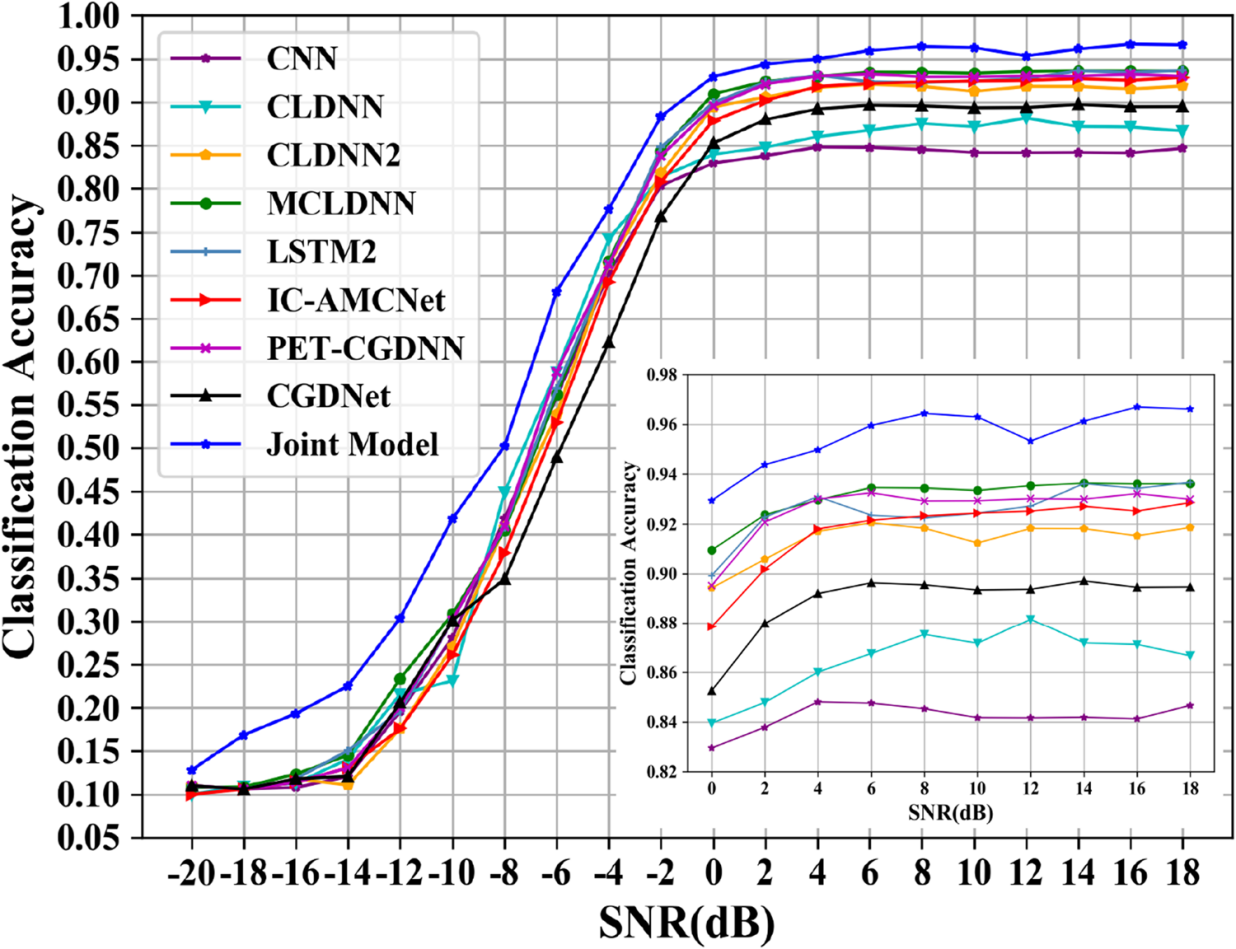
Classification accuracy of different networks on the RML2016.10b dataset.

Similarly, we plot a comparison diagram of the overall accuracy of signal recognition between the joint model and the benchmark network under different SNRs. As can be seen from the figure, the joint model has better recognition performance. When the SNR is 16 dB, the recognition accuracy reaches 96.69%, which is 3.22% higher than the MCLDNN network. As shown in Fig. 18.

**Figure 19:**
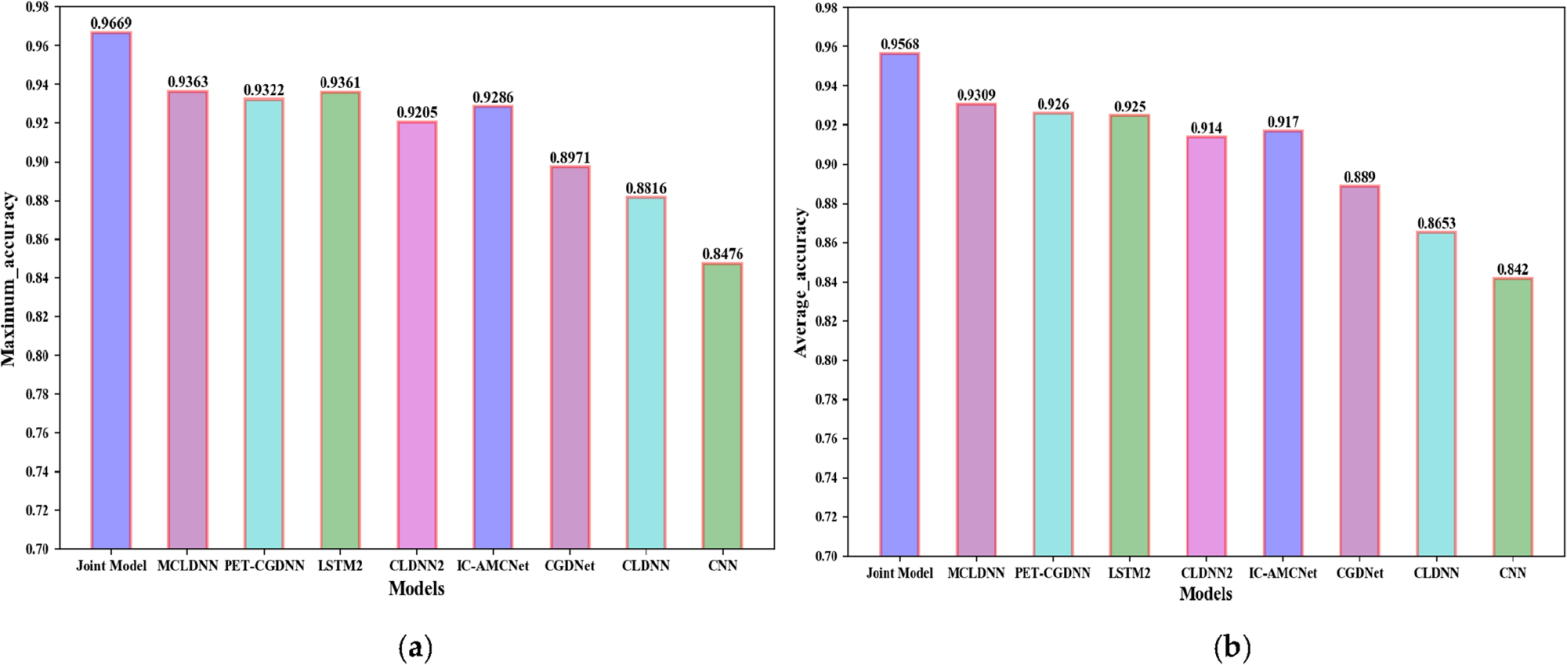
Accuracy comparison chart of the nine models on the RML2016.10b dataset. (**a**) The maximum accuracy; (**b**) The average accuracy.

The recognition accuracies obtained by the nine networks are visually represented by a histogram, as shown in Fig. 19.

## Conclusion

In this paper, we propose a time–frequency domain joint AMR model that combines two deep learning networks, the MCLDNN and BiGRU3, to identify different modulated signals commonly used in wireless communication. Introduce FFT to obtain amplitude and phase feature information of AM-DSB and WBFM in the frequency domain. And form a new two-class dataset called DW based on the amplitude and phase characteristics. Built a novel deep learning network, BiGRU3, that can accurately extract amplitude spectrum and phase spectrum features in the frequency domain. The MCLDNN network can accurately separate DW signals from other types of signals. Then, the DW dataset is accurately classified by combining FFT and BiGRU3 network. The results show that the joint AMR model has better recognition performance than the baseline network. On the RML2016.10a dataset, the binary classification accuracy is improved by 17%. And the overall recognition accuracy of the model is enhanced by 2.67%, reaches 94.94%. On the RML2016.10b dataset, the binary classification accuracy is improved by 18.2%. And the overall recognition accuracy of the model is enhanced by 3.22%, reaches 96.69%. The recognition performance reaches a better level, and the research in this paper has a promising frontier.

This study provides a new deep learning architecture in spatial cognitive radio, which combines multiple neural networks flexibly and incorporates the idea of multi- domain fusion to better achieve accurate recognition of various signals in complex radio environment. This article uses two datasets, the RML2016.10a and RML2016.10b, which have limitation in the number of modulation categories. Therefore, the next step can consider using the RML2018.01a and HisarMod2019.1 datasets^42^ to specifically design corresponding joint recognition networks for the similarity between more categories of signals.

In future research, in addition to designing joint recognition model to recognize more types of signals, the attention mechanism module can be introduced in the deep learning model to further improve the classification performance of the joint modulation recognition network for signals^43,44^. Also, in future work, how to use machine learning knowledge to enhance the security of data processing in wireless communication systems is also a challenging task^45–47^.

## Data Availability

The data in this manuscript is available upon reasonable request from the corresponding authors.
